# 4-Chloro-*N*′-[(3*Z*)-2-oxo-2,3-di­hydro-1*H*-indol-3-yl­idene]benzohydrazide

**DOI:** 10.1107/S1600536813033345

**Published:** 2013-12-14

**Authors:** Shaaban K. Mohamed, Joel T. Mague, Mehmet Akkurt, Abdel-Aal M. Jaber, Mustafa R. Albayati

**Affiliations:** a Chemistry and Environmental Division, Manchester Metropolitan University, Manchester M1 5GD, England; b Chemistry Department, Faculty of Science, Minia University, 61519 El-Minia, Egypt; c Department of Chemistry, Tulane University, New Orleans, LA 70118, USA; d Department of Physics, Faculty of Sciences, Erciyes University, 38039 Kayseri, Turkey; e Chemistry Department, Faculty of Science, Assiut University, Assiut, Egypt; f Kirkuk University, College of Science, Department of Chemistry, Kirkuk, Iraq

## Abstract

In the title compound, C_15_H_10_ClN_3_O_2_, the benzene ring is slightly twisted out of the plane of the 2,3-di­hydro-1*H*-indole ring system (r.m.s. deviation = 0.007 Å), forming a dihedral angle of 7.4 (3)°. An intra­molecular N—H⋯O hydrogen bond forms a six-membered ring. In the crystal, mol­ecules are linked *via* N—H⋯O and C—H⋯O hydrogen bonds, forming layers alternately perpendicular to [011] and [0-11].

## Related literature   

For the diverse bio-activities of acid hydrazides and their condensed products, see: Adekunle *et al.* (2012 ▶); Al-Assar *et al.* (2002 ▶); Dharmaraj *et al.* (2001 ▶); Jain & Vederas (2004 ▶); Jeeworth *et al.* (2000 ▶); Scozzafava *et al.* (2001 ▶); Siddappa *et al.* (2008 ▶); Strappaghetti *et al.* (2006 ▶).
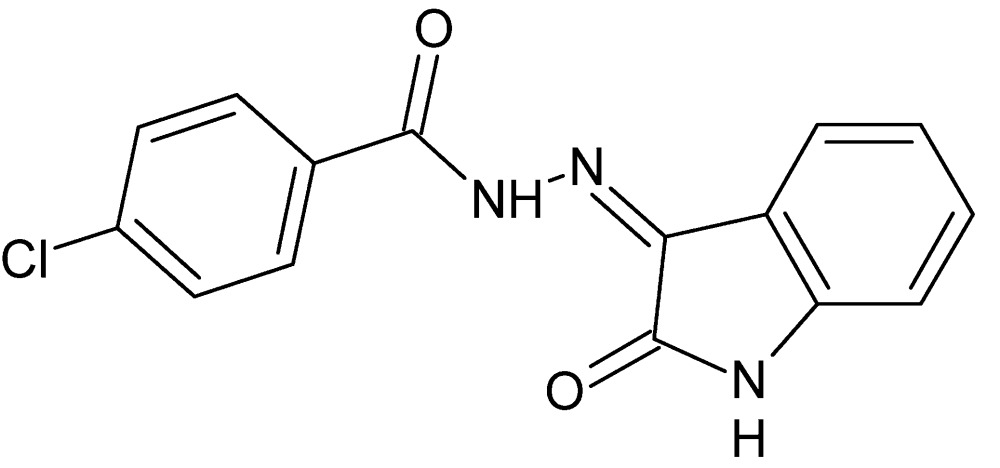


## Experimental   

### 

#### Crystal data  

C_15_H_10_ClN_3_O_2_
*M* *_r_* = 299.71
Orthorhombic, 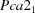

*a* = 31.0359 (12) Å
*b* = 5.2549 (3) Å
*c* = 7.8730 (4) Å
*V* = 1284.01 (11) Å^3^
*Z* = 4
Cu *K*α radiation
μ = 2.72 mm^−1^
*T* = 102 K
0.22 × 0.17 × 0.01 mm

#### Data collection  

Bruker D8 VENTURE PHOTON 100 CMOS diffractometer
Absorption correction: multi-scan (*SADABS*; Bruker, 2013 ▶) *T*_min_ = 0.75, *T*_max_ = 0.97
4190 measured reflections
1596 independent reflections
1434 reflections with *I* > 2σ(*I*)
*R*_int_ = 0.109

#### Refinement  

*R*[*F* ^2^ > 2σ(*F* ^2^)] = 0.059
*wR*(*F* ^2^) = 0.152
*S* = 1.16
1596 reflections
193 parameters
3 restraints
H atoms treated by a mixture of independent and constrained refinement
Δρ_max_ = 0.30 e Å^−3^
Δρ_min_ = −0.37 e Å^−3^
Absolute structure: Flack (1983 ▶), 478 Friedal pairs (44% coverage)
Absolute structure parameter: −0.06 (5)

### 

Data collection: *APEX2* (Bruker, 2013 ▶); cell refinement: *SAINT* (Bruker, 2013 ▶); data reduction: *SAINT*; program(s) used to solve structure: *SHELXS97* (Sheldrick, 2008 ▶); program(s) used to refine structure: *SHELXL97* (Sheldrick, 2008 ▶); molecular graphics: *ORTEP-3 for Windows* (Farrugia, 2012 ▶); software used to prepare material for publication: *PLATON* (Spek, 2009 ▶).

## Supplementary Material

Crystal structure: contains datablock(s) global, I. DOI: 10.1107/S1600536813033345/su2672sup1.cif

Structure factors: contains datablock(s) I. DOI: 10.1107/S1600536813033345/su2672Isup2.hkl

Supporting information file. DOI: 10.1107/S1600536813033345/su2672Isup3.cml

Additional supporting information:  crystallographic information; 3D view; checkCIF report

## Figures and Tables

**Table 1:** Hydrogen-bond geometry (Å, °)

*D*—H⋯*A*	*D*—H	H⋯*A*	*D*⋯*A*	*D*—H⋯*A*
N1—H1⋯O2	0.90 (5)	1.87 (6)	2.685 (9)	151 (4)
N3—H3*A*⋯O1^i^	0.91	1.98	2.798 (8)	149
C11—H11⋯O1^i^	0.95	2.55	3.218 (10)	128
C14—H14⋯O2^ii^	0.95	2.29	3.233 (9)	172

Symmetry codes: (i) 
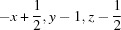
; (ii) 
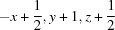
.

**Fig. 1.**
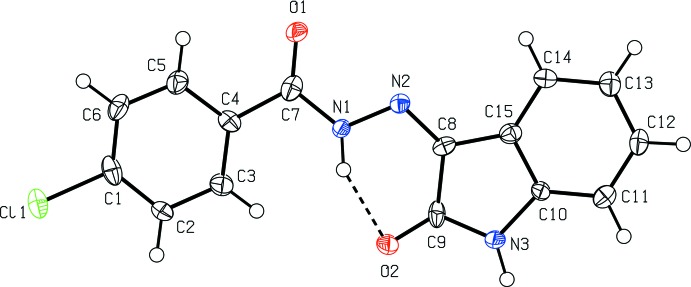
The molecular structure of the title molecule, with atom labelling. Displacement ellipsoids are drawn at the 50% probability level. The intramolecular N-H···O hydrogen bond is shown as a dashed line (see Table 1 for details).

**Fig. 2.**
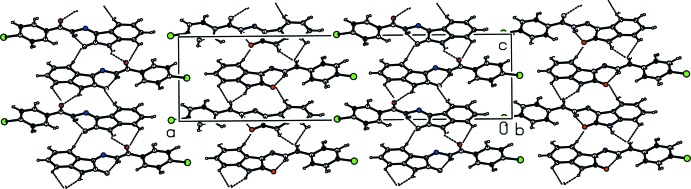
A view along the b axis of the crystal packing of the title compound. The hydrogen bonds are shown as a dashed lines (see Table 1 for details).

## supplementary crystallographic information

### 1. Comment

Hydrazide compounds and their condensation products exhibit a wide range of biological activities such as anti-cancer (Strappaghetti *et al.*, 2006), anti-depressant (Al-Assar *et al.*, 2002), anti-HIV (Adekunle *et al.*, 2012), analgesic-anti-inflammatory (Jain & Vederas, 2004), bactericidal (Jeeworth *et al.*, 2000), leishmanicidal (Scozzafava *et al.*, 2001), anti-helmintic (Dharmaraj *et al.*, 2001) and anti-tuberculosis activities (Siddappa *et al.*, 2008). Based on such findings and continuing to our on-going study of the synthesis of bio-active heterocyclic compounds, we herein report on the synthesis and crystal structure of the title compound.

In the title compound, Fig. 1, the intramolecular N1—H1···O2 hydrogen bond forms a pseudo-six-membered ring. The nine non-H ring atoms of the fused five- and six-membered ring system are almost coplanar (r.m.s. deviation = 0.007 Å). The benzene ring and the 2,3-dihydro-1*H*-indole ring system make a dihedral angle of 7.4 (3)° .

In the crystal structure, molecular layers formed by N—H···O and C-H···O hydrogen bonds are alternately perpendicular to [011] and to [0–11] directions (Table 1 and Fig. 2).

### 2. Experimental

A mixture of 1 mmol (170.6 mg) 4-chlorobenzohydrazidean and 1 mmol (147 mg) 1*H*-indole-2,3-dione in 25 ml ethanol with few drops of glacial acetic acid was refluxed for 5h. The solid formed was collected and recrystallized from DMF to furnish the title compound as yellow crystals suitable for X-ray analysis [*M*.p. 558 K].

### 3. Refinement

The NH H atoms (H1 on N1) was located in a difference Fourier map and refined with a distance restraint: N1—H1 = 0.90 (5) Å with *U*_iso_(H) = 1.2*U*_eq_(N). The remaining H atoms were placed in calculated positions and refined using a riding model approximation: C—H = 0.95 Å and N—H = 0.91 Å with *U*_iso_(H) = 1.2*U*_eq_(C, N). The small proportion of reflections observed is a result of the rather poor quality of the very thin crystals obtained.

### Crystal data

**Table:**

C_15_H_10_ClN_3_O_2_	*F*(000) = 616
*M**_r_* = 299.71	*D*_x_ = 1.550 Mg m^−^^3^
Orthorhombic, *P**c**a*2_1_	Cu *K*α radiation, λ = 1.54178 Å
Hall symbol: P 2c -2ac	Cell parameters from 3290 reflections
*a* = 31.0359 (12) Å	θ = 2.9–68.5°
*b* = 5.2549 (3) Å	µ = 2.72 mm^−^^1^
*c* = 7.8730 (4) Å	*T* = 102 K
*V* = 1284.01 (11) Å^3^	Plate, yellow
*Z* = 4	0.22 × 0.17 × 0.01 mm

### Data collection

**Table:**

Bruker D8 VENTURE PHOTON 100 CMOS diffractometer	1596 independent reflections
Radiation source: INCOATEC IµS micro–focus source	1434 reflections with *I* > 2σ(*I*)
Mirror monochromator	*R*_int_ = 0.109
Detector resolution: 10.4167 pixels mm^-1^	θ_max_ = 68.5°, θ_min_ = 5.7°
ω scans	*h* = −32→37
Absorption correction: multi-scan (*SADABS*; Bruker, 2013)	*k* = −5→5
*T*_min_ = 0.75, *T*_max_ = 0.97	*l* = −8→9
4190 measured reflections	

### Refinement

**Table:**

Refinement on *F*^2^	Secondary atom site location: inferred from neighbouring sites
Least-squares matrix: full	H atoms treated by a mixture of independent and constrained refinement
*R*[*F*^2^ > 2σ(*F*^2^)] = 0.059	W = 1/[Σ^2^(*F*_o_^2^) + (0.0459*P*)^2^ + 3.7436*P*] where *P* = (*F*_o_^2^ + 2*F*_c_^2^)/3
*wR*(*F*^2^) = 0.152	(Δ/σ)_max_ < 0.001
*S* = 1.16	Δρ_max_ = 0.30 e Å^−^^3^
1596 reflections	Δρ_min_ = −0.37 e Å^−^^3^
193 parameters	Absolute structure: Flack (1983), 478 Friedal pairs (44% coverage)
3 restraints	Absolute structure parameter: −0.06 (5)
Primary atom site location: difference Fourier map	

### Special details

**Table:**

Geometry. Bond distances, angles *etc*. have been calculated using the rounded fractional coordinates. All su's are estimated from the variances of the (full) variance-covariance matrix. The cell e.s.d.'s are taken into account in the estimation of distances, angles and torsion angles
Refinement. Refinement on *F*^2^ for ALL reflections except those flagged by the user for potential systematic errors. Weighted *R*-factors *wR* and all goodnesses of fit *S* are based on *F*^2^, conventional *R*-factors *R* are based on *F*, with *F* set to zero for negative *F*^2^. The observed criterion of *F*^2^ > σ(*F*^2^) is used only for calculating -*R*-factor-obs *etc*. and is not relevant to the choice of reflections for refinement. *R*-factors based on *F*^2^ are statistically about twice as large as those based on *F*, and *R*-factors based on ALL data will be even larger.

### Fractional atomic coordinates and isotropic or equivalent isotropic displacement parameters (Å^2^)

**Table:**

	*x*	*y*	*z*	*U*_iso_*/*U*_eq_	
Cl1	−0.02432 (5)	0.0784 (4)	0.5130 (3)	0.0317 (6)	
O1	0.15220 (15)	0.7554 (12)	0.7066 (7)	0.0247 (19)	
O2	0.21783 (16)	0.0289 (11)	0.3951 (7)	0.0240 (19)	
N1	0.18451 (17)	0.4151 (14)	0.5756 (8)	0.0187 (19)	
N2	0.22478 (17)	0.5046 (12)	0.6135 (8)	0.0173 (19)	
N3	0.29242 (19)	0.0513 (13)	0.4056 (8)	0.0203 (19)	
C1	0.0263 (2)	0.2224 (17)	0.5393 (10)	0.026 (3)	
C2	0.0616 (2)	0.1072 (16)	0.4604 (10)	0.023 (2)	
C3	0.1021 (2)	0.2172 (17)	0.4862 (10)	0.026 (3)	
C4	0.1069 (2)	0.4273 (16)	0.5882 (9)	0.020 (2)	
C5	0.0705 (2)	0.5395 (17)	0.6625 (10)	0.026 (3)	
C6	0.0301 (2)	0.4340 (18)	0.6391 (11)	0.028 (3)	
C7	0.1495 (2)	0.5554 (18)	0.6291 (10)	0.024 (3)	
C8	0.2560 (2)	0.3623 (15)	0.5494 (9)	0.018 (2)	
C9	0.2517 (2)	0.1344 (16)	0.4425 (10)	0.022 (2)	
C10	0.3234 (2)	0.2196 (15)	0.4808 (9)	0.018 (2)	
C11	0.3677 (2)	0.2028 (16)	0.4744 (10)	0.022 (2)	
C12	0.3910 (2)	0.3895 (16)	0.5590 (10)	0.025 (3)	
C13	0.3700 (2)	0.5837 (17)	0.6480 (10)	0.025 (3)	
C14	0.3253 (2)	0.5978 (15)	0.6566 (9)	0.019 (3)	
C15	0.3018 (2)	0.4074 (16)	0.5723 (9)	0.021 (3)	
H1	0.1866 (10)	0.264 (9)	0.523 (10)	0.0220*	
H2	0.05820	−0.04030	0.39190	0.0280*	
H3	0.12660	0.14550	0.43240	0.0310*	
H3A	0.30090	−0.06130	0.32440	0.0250*	
H5	0.07370	0.68880	0.72940	0.0320*	
H6	0.00550	0.50730	0.69140	0.0340*	
H11	0.38160	0.06920	0.41470	0.0270*	
H12	0.42160	0.38530	0.55640	0.0290*	
H13	0.38670	0.70950	0.70410	0.0300*	
H14	0.31130	0.73060	0.71710	0.0230*	

### Atomic displacement parameters (Å^2^)

**Table:**

	*U*^11^	*U*^22^	*U*^33^	*U*^12^	*U*^13^	*U*^23^
Cl1	0.0189 (8)	0.0414 (13)	0.0349 (11)	−0.0052 (7)	−0.0031 (9)	0.0024 (11)
O1	0.022 (3)	0.030 (4)	0.022 (3)	0.004 (2)	−0.001 (2)	−0.009 (3)
O2	0.023 (3)	0.030 (4)	0.019 (3)	−0.002 (2)	−0.001 (2)	−0.007 (3)
N1	0.015 (3)	0.024 (4)	0.017 (3)	0.003 (2)	−0.002 (2)	−0.006 (3)
N2	0.019 (3)	0.021 (4)	0.012 (3)	0.000 (2)	0.002 (2)	0.000 (3)
N3	0.020 (3)	0.024 (4)	0.017 (3)	0.001 (3)	−0.001 (3)	−0.007 (3)
C1	0.020 (3)	0.040 (6)	0.019 (4)	−0.006 (3)	−0.008 (3)	0.009 (4)
C2	0.023 (3)	0.023 (5)	0.024 (4)	−0.003 (3)	−0.004 (3)	−0.006 (4)
C3	0.026 (4)	0.033 (5)	0.019 (4)	−0.001 (3)	−0.002 (4)	−0.005 (4)
C4	0.025 (4)	0.025 (5)	0.010 (3)	−0.004 (3)	−0.003 (3)	0.002 (3)
C5	0.022 (4)	0.039 (6)	0.018 (4)	0.003 (3)	0.001 (3)	−0.005 (4)
C6	0.021 (4)	0.040 (6)	0.023 (4)	0.007 (3)	0.005 (3)	−0.001 (4)
C7	0.022 (4)	0.036 (6)	0.015 (4)	0.006 (3)	0.001 (3)	0.003 (4)
C8	0.025 (3)	0.019 (5)	0.009 (4)	0.002 (3)	0.005 (3)	0.002 (3)
C9	0.013 (3)	0.038 (5)	0.016 (4)	0.001 (3)	−0.002 (3)	0.005 (4)
C10	0.017 (3)	0.025 (5)	0.013 (4)	0.000 (3)	−0.003 (3)	−0.004 (3)
C11	0.025 (3)	0.022 (5)	0.020 (4)	0.006 (3)	0.000 (3)	0.004 (4)
C12	0.017 (3)	0.034 (6)	0.023 (5)	0.002 (3)	0.000 (3)	0.003 (4)
C13	0.022 (4)	0.034 (6)	0.018 (4)	−0.001 (3)	−0.001 (3)	0.001 (4)
C14	0.026 (4)	0.016 (5)	0.016 (4)	0.000 (3)	−0.001 (3)	0.001 (3)
C15	0.024 (4)	0.022 (5)	0.018 (4)	0.002 (3)	−0.001 (3)	−0.001 (3)

### Geometric parameters (Å, º)

**Table:**

Cl1—C1	1.756 (7)	C8—C9	1.470 (11)
O1—C7	1.218 (11)	C8—C15	1.452 (9)
O2—C9	1.246 (9)	C10—C15	1.394 (11)
N1—N2	1.368 (8)	C10—C11	1.379 (9)
N1—C7	1.379 (10)	C11—C12	1.389 (11)
N2—C8	1.324 (9)	C12—C13	1.399 (11)
N3—C9	1.368 (9)	C13—C14	1.391 (9)
N3—C10	1.434 (9)	C14—C15	1.405 (11)
N1—H1	0.90 (5)	C2—H2	0.9500
N3—H3A	0.9100	C3—H3	0.9500
C1—C6	1.367 (13)	C5—H5	0.9500
C1—C2	1.397 (10)	C6—H6	0.9500
C2—C3	1.398 (10)	C11—H11	0.9500
C3—C4	1.373 (12)	C12—H12	0.9500
C4—C5	1.402 (10)	C13—H13	0.9500
C4—C7	1.518 (10)	C14—H14	0.9500
C5—C6	1.383 (10)		
			
N2—N1—C7	118.0 (7)	N3—C10—C15	109.1 (6)
N1—N2—C8	113.0 (6)	C11—C10—C15	123.0 (7)
C9—N3—C10	109.6 (6)	N3—C10—C11	127.9 (7)
C7—N1—H1	132 (2)	C10—C11—C12	117.2 (7)
N2—N1—H1	110 (2)	C11—C12—C13	120.9 (6)
C9—N3—H3A	129.00	C12—C13—C14	121.9 (7)
C10—N3—H3A	120.00	C13—C14—C15	117.2 (7)
Cl1—C1—C6	119.7 (5)	C8—C15—C14	133.1 (7)
C2—C1—C6	122.7 (6)	C10—C15—C14	119.9 (6)
Cl1—C1—C2	117.5 (6)	C8—C15—C10	106.9 (6)
C1—C2—C3	117.5 (7)	C1—C2—H2	121.00
C2—C3—C4	121.0 (7)	C3—C2—H2	121.00
C3—C4—C5	119.6 (6)	C2—C3—H3	119.00
C3—C4—C7	125.1 (6)	C4—C3—H3	120.00
C5—C4—C7	115.3 (7)	C4—C5—H5	120.00
C4—C5—C6	120.4 (8)	C6—C5—H5	120.00
C1—C6—C5	118.7 (7)	C1—C6—H6	121.00
O1—C7—C4	123.3 (6)	C5—C6—H6	121.00
N1—C7—C4	112.6 (7)	C10—C11—H11	121.00
O1—C7—N1	124.1 (6)	C12—C11—H11	122.00
N2—C8—C9	127.7 (6)	C11—C12—H12	120.00
N2—C8—C15	125.2 (7)	C13—C12—H12	120.00
C9—C8—C15	107.0 (6)	C12—C13—H13	119.00
N3—C9—C8	107.3 (6)	C14—C13—H13	119.00
O2—C9—N3	125.0 (7)	C13—C14—H14	121.00
O2—C9—C8	127.7 (6)	C15—C14—H14	122.00
			
C7—N1—N2—C8	−177.0 (7)	C5—C4—C7—N1	170.3 (7)
N2—N1—C7—O1	0.7 (12)	C4—C5—C6—C1	−1.5 (13)
N2—N1—C7—C4	−176.6 (6)	N2—C8—C9—O2	−1.7 (14)
N1—N2—C8—C9	2.1 (11)	N2—C8—C9—N3	179.7 (7)
N1—N2—C8—C15	−178.5 (7)	C15—C8—C9—O2	178.8 (8)
C10—N3—C9—O2	179.9 (7)	C15—C8—C9—N3	0.1 (8)
C10—N3—C9—C8	−1.4 (8)	N2—C8—C15—C10	−178.3 (7)
C9—N3—C10—C11	179.7 (8)	N2—C8—C15—C14	−1.6 (14)
C9—N3—C10—C15	2.2 (9)	C9—C8—C15—C10	1.2 (8)
Cl1—C1—C2—C3	−177.9 (6)	C9—C8—C15—C14	177.9 (8)
C6—C1—C2—C3	−0.2 (12)	N3—C10—C11—C12	−179.6 (7)
Cl1—C1—C6—C5	177.9 (7)	C15—C10—C11—C12	−2.4 (12)
C2—C1—C6—C5	0.3 (13)	N3—C10—C15—C8	−2.1 (8)
C1—C2—C3—C4	1.3 (12)	N3—C10—C15—C14	−179.3 (7)
C2—C3—C4—C5	−2.5 (12)	C11—C10—C15—C8	−179.7 (7)
C2—C3—C4—C7	177.8 (8)	C11—C10—C15—C14	3.1 (12)
C3—C4—C5—C6	2.6 (12)	C10—C11—C12—C13	0.7 (12)
C7—C4—C5—C6	−177.7 (8)	C11—C12—C13—C14	0.4 (12)
C3—C4—C7—O1	172.7 (8)	C12—C13—C14—C15	0.2 (12)
C3—C4—C7—N1	−10.0 (11)	C13—C14—C15—C8	−178.2 (8)
C5—C4—C7—O1	−7.0 (12)	C13—C14—C15—C10	−1.9 (11)

### Hydrogen-bond geometry (Å, º)

**Table:**

*D*—H···*A*	*D*—H	H···*A*	*D*···*A*	*D*—H···*A*
N1—H1···O2	0.90 (5)	1.87 (6)	2.685 (9)	151 (4)
N3—H3*A*···O1^i^	0.91	1.98	2.798 (8)	149
C11—H11···O1^i^	0.95	2.55	3.218 (10)	128
C14—H14···O2^ii^	0.95	2.29	3.233 (9)	172

Symmetry codes: (i) −*x*+1/2, *y*−1, *z*−1/2; (ii) −*x*+1/2, *y*+1, *z*+1/2.
